# Thrombocytopenia in Chronic Liver Disease: Challenges and Treatment Strategies

**DOI:** 10.7759/cureus.16342

**Published:** 2021-07-12

**Authors:** Shreya Desai, Anita Subramanian

**Affiliations:** 1 Internal Medicine, Rosalind Franklin University of Medicine and Science, North Chicago, USA; 2 Internal Medicine, Campbell University, Lilington, USA; 3 Internal Medicine, Ross University School of Medicine, Barbados, BRB

**Keywords:** cirrhosis, thrombocytopenia, tips, blood transfusion, chronic liver disease (cld), tpo receptor agonist

## Abstract

Thrombocytopenia is a common hematologic complication seen in patients with chronic liver disease (CLD). The pathophysiology of thrombocytopenia in CLD is multifactorial, primarily stemming from platelet sequestration and decreased platelet production. This review focuses on the pathophysiology and current treatment options in the treatment of thrombocytopenia in chronic liver disease. While platelet transfusions are the gold standard of treatment, considerations ought to be given to CLD patients who can benefit from transjugular intrahepatic portosystemic shunt and splenic artery embolization. Finally, the recent approval of thrombopoietin receptor agonists for use in CLD patients paves a way for a safe and effective alternative method of improving platelet levels and reducing the need for recurrent platelet transfusions.

## Introduction and background

Thrombocytopenia is a common hematological abnormality in patients with chronic liver disease (CLD). Thrombocytopenia is defined as a platelet count less than 150,000/μL [1]. Up to 76% of patients with chronic liver disease are thought to have some degree of thrombocytopenia which higher incidence observed in patients with cirrhosis [2]. The clinical significance of mild thrombocytopenia (75,000/μL- 150,000/μL) is minimal and usually does not require treatment [2]. However, severe thrombocytopenia (<50,000/μL) can often complicate the management of patients with chronic liver disease due to increased risk of bleeding. This review focuses on the pathophysiology and the current treatment strategies for the treatment of thrombocytopenia in chronic liver disease. 

## Review

Pathophysiology of thrombocytopenia in chronic liver disease

The pathophysiology of thrombocytopenia in chronic liver disease has been attributed to multiple factors. Historically, attributed to hypersplenism due to increased splenic vein pressure, improved understanding of thrombocytopenia in chronic liver disease has demonstrated a multifactorial mechanism [3]. This can be broken down into two broader causes: decreased production of platelets, and increased destruction. The decreased production of platelets can be attributed to both a reduction in thrombopoietin production in the liver and reduced bone marrow production of platelets secondary to several inhibiting causes [3]. Hepatic production of thrombopoietin plays a pivotal role in thrombopoiesis. Thrombopoietin (TPO), in turn, binds to the c-mpl receptor on megakaryocytes regulating their differentiation into platelets [3]. There is a recognized direct correlation between chronic liver disease and a decrease in circulating TPO and it has been suggested that monitoring circulating TPO levels can evaluate the degree of thrombocytopenia in chronic liver disease [4]. Additionally, reduced bone marrow production most commonly secondary to viruses, alcohol use, iron overload, or medications contributes to reduced platelet production [3]. Hepatitis C and B, most frequently associated with liver cirrhosis, inhibits growth and differentiation of human bone marrow progenitor cells resulting in direct bone marrow suppression [3]. Alcohol use, a major contributor to CLD, both reduces platelet life span and leads to ineffective megakaryopoiesis [3]. CLD also notably impairs the liver’s ability to esterify cholesterol resulting in spur cell hemolytic anemia which causes iron overload. Increased iron levels decrease prohepcidin expression and increase iron absorption, causing platelets to have a diminished response to EPO. Medications like interferon (used to treat Hepatitis B/C) are associated with impaired thrombopoiesis as well.

Furthermore, increased destruction of platelets in CLD is ascribed to splenic sequestration and immune-mediated destruction [3,5]. In chronic liver disease, the spleen is enlarged because of portal hypertension, increasing pooling of platelets. Conversely an inverse relationship has been observed between spleen size and platelet count. Additionally, immune-mediated destruction plays a large role in platelet destruction in chronic liver disease. Most associated causes include autoimmune conditions such as immune-mediated thrombocytopenia, hepatitis C or sepsis [4]. In autoimmune or viral disease, antiplatelet antibodies are formed resulting in platelet destruction. Sepsis in cirrhotic patients is triggered by endotoxemia accelerating platelet consumption through the stimulation of B-cell activity and production of IgG [3,5]. Although thrombocytopenia in end-stage liver disease is the most commonly documented abnormality at this time, there is no direct evidence correlating platelet count and disease severity. While studies have shown that a progressive decline in liver function does directly correlate to a lower platelet count, there are no formal guidelines for indicators of disease severity [6]. However, since individuals with cirrhosis or chronic liver disease often require frequent interventions or medications, those with severe thrombocytopenia are often associated with poorer prognosis due to delay to treatment [4,7,8].

Currently, no guidelines exist for laboratory monitoring in thrombocytopenia without evidence of bleeding. While it is common practice to monitor platelet levels and intervene prior to planned treatments or procedures, these are not formally indicated but rather more clinician-based [2, 8]. Harrison explored further the concept of reliable monitoring, finding no strong evidence towards the predictability of monitoring coagulation tests and bleeding risk; monitoring is often associated with increased prophylactic management in unnecessary cases [7]. In general, monitoring INR or platelet count as a tool to assess the degree of coagulopathy is not considered the best-practice in literature. Rather, the current practice for the management of thrombocytopenia in chronic liver disease prior to interventions is clinician and procedure-based.

Platelet transfusion

The gold standard for management of thrombocytopenia has been widely accepted as platelet transfusions. However, with this treatment comes many disadvantages and unclear beneficial effects that are steering clinicians away from its frequent use [8]. Currently, no clear guidelines exist for cutoffs regarding prophylactic transfusions. Rather, decisions are commonly made based on the type of procedure and by the clinician’s discretion. Additionally, risks of transfusion include a higher rate of infection, febrile transfusion reactions, allergic/hypersensitivity reactions, transfusion-related acute lung injury, transfusion-associated circulatory overload, hemolysis secondary to ABO incompatibility, and graft vs host disease [7]. Harrison explains further, stating that a lack of sufficient evidence currently exists to attempt optimization of platelet count or INR in prophylactic management [6]. The long-term risk of alloimmunization associated with repeated platelet transfusions causing platelet refractoriness can threaten any future need for emergent management [7]. At this time, common practice indicates individuals with thrombocytopenia and CLD undergo prophylactic measures depending on the degree of disease and risk of bleeding in procedures. Higher-risk procedures such as intracranial or spinal procedures typically suggest a goal platelet count of >100,000/µL, while for lower-risk procedures such as paracentesis or EGD/colonoscopy, a goal of >20,000/µL is considered acceptable [7]. 

Transjugular intrahepatic portosystemic shunt (TIPS)

Transjugular intrahepatic portosystemic shunt (TIPS) is a minimally invasive treatment option for patients with variceal bleeding and refractory ascites. The procedure works by placing a small wire-mesh coil into a liver vein where a stent is expanded. This forms a shunt that bypasses the liver, in turn reducing pressure in the portal vein [9]. This procedure has been shown to effectively reduce portal hypertension and the complications associated with it [9]. Its effect on thrombocytopenia has not been widely studied and few studies to date have been published. However, those that have been studied have documented a significant increase in platelet count in post-TIPS liver cirrhosis patients.

While few studies at this time have been performed to determine the efficacy of TIPS on platelet count, the majority of those published have shown significant improvement in thrombocytopenia in CLD post-procedure. In 2017, Massoud et al. assessed the effect of TIPS on thrombocytopenia by monitoring 74 patients with liver cirrhosis who were referred for TIPS [9]. Platelet counts were measured three different times over a three-month period prior to and following the placement of TIPS. They set a significant increase in platelet count as 20% or higher from the pre-TIPS value. Results of the study showed that 46% of individuals who underwent TIPS showed a significant increase in platelet count, with an average increase of 22%. Most notably, patients with severe thrombocytopenia showed the greatest response to TIPS: 8 out of 11 patients had a significant increase of on average 55% in platelet count. The study found that TIPS may improve thrombocytopenia in liver cirrhosis with the most significant benefit shown in patients with severe thrombocytopenia (<50,000/µL) [9]. The results of this study corroborated evidence from five other past studies showing similar benefits of TIPS on thrombocytopenia in chronic liver disease. Alvarez et al was a case series performed in 1996 that followed 11 patients in the 12-month follow-up period after TIPS [10]. They concluded a statistically significant improvement in thrombocytopenia and hemodynamic improvement.

Of the few studies published on the effect of TIPS on thrombocytopenia, less than half have documented no significant improvement in platelet count post-TIPS procedure. One example is a retrospective study performed by Karasu and colleagues in 2001 that reviewed 58 patients who underwent the TIPS procedure. They concluded an unpredictable effect on platelet counts in cirrhotic patients with no observable consistent increase in platelet volume [11]. However, of note, this study only documented one pre-TIPS platelet count reading and one reading post-TIPS and likely based their conclusions on insufficient data evidence as it is widely acknowledged that platelet counts can greatly vary daily even in a healthy individual. Another study that concluded no improvement was a 1998 retrospective study performed by Jabbour et al. This study observed 62 patients who underwent TIPS procedure and found no significant change in platelet count post-procedure [12]. Notably, this study defined a successful increase in platelet count as > 100,000/µL, rather than an observed percentage increase from pre-TIPS values. So, while a significant increase in severe thrombocytopenia was noted (20,000-30,000/µL to 80,000-90,000/µL) they were considered not responsive to therapy as they remained under 100,000/µL [12]. Thus, while not specifically indicated for the treatment of thrombocytopenia, TIPS appears to be a relatively safe and effective method of improving thrombocytopenia in patients with chronic liver disease.

Splenic artery embolization

Splenomegaly is a frequent sequela of cirrhosis and is associated with decreased hematologic indices including thrombocytopenia. As such, spleen interventions may effectively increase hematologic values in patients with cirrhosis and concurrent hypersplenism. Currently, two methods of interventions are considered: splenectomy and partial splenic embolization. Splenectomy remains a risky procedure with significant associated morbidity [13]. While many patients with advanced cirrhosis may not be candidates for the surgery, other major risks include the risk of portal vein thrombosis, bacterial infection, and rarely, liver failure [13]. Alternatively, partial splenic artery embolization (PSE) has been proposed as a safer alternative treatment to hypersplenism with significant improvement in leukopenia and thrombocytopenia in the immediate months following the procedure [14].

Following PSE, a substantial improvement is noted in thrombocytopenia. However, the duration of improvement and optimal volume of splenic infarction varies widely. Various studies have used 50-80% infarcted splenic volume as a target following PSE [14-16]. A study of 13 patients with cirrhotic hypersplenism who underwent 80% PSE had marked improvement in platelet counts with sustained effect up to 36 months following the procedure [15]. Another retrospective analysis of cancer patients with hypersplenism who underwent PSE showed that the percentage volume of infarcted splenic tissue linearly correlated with the magnitude of platelet increase (p= 0.001). Following PSE, 41% of patients did not have a recurrence of thrombocytopenia for the duration of their survival [17]. While encouraging, the data from this study may not indicate similar outcomes in cirrhosis patients potentially due to increased lifespan compared to a cohort of cancer patients. Indeed, studies have shown the occurrence of recurrent thrombocytopenia in cirrhotic patients either due to revascularization or reperfusion of the embolized splenic arteries [18].

While the increased volume of infarcted tissue correlates to an increased magnitude of improvement in hematologic indices, it also carries an increased risk of complications. The most common complication of PSE is post-embolization syndrome, which consists of a myriad of symptoms including fever, nausea, vomiting, and pain. Portal vein thrombosis may also occur following PSE, although it is seen in decreased frequency compared to splenectomy [14]. There is an increased risk of splenic abscess following PSE [19]. Child-Pugh class C and percentage of infarcted splenic volume have been found to be associated with a higher likelihood of complications following PSE [16-18]. Despite complications, the longer duration of improved blood counts may make PSE an appealing treatment strategy in some patients with severe thrombocytopenia. However, the risk of complications prevents its widespread use and warrants further research. An interventional trial of 20 patients with refractory thrombocytopenia due to cirrhosis is currently evaluating the use of Yttrium-90 (90Y) radioembolization for management of thrombocytopenia (NCT03059030).

Interestingly, some have proposed total splenic artery embolization (TSAE) as a safer alternative to PSE in patients with cirrhotic hypersplenism. A trial of 61 patients evaluating the effectiveness of TSAE versus PSE showed significantly higher elevations in white blood cell and platelet counts following TSAE (p= 0.001) with significantly fewer complications and shorter hospital stay (p= 0.001) [20]. 

Thrombopoietin receptor agonists

Thrombopoietin (TPO) is a peptide cytokine primarily produced by the liver and regulates platelet production. TPO binds to TPO receptors (c-mpl) on platelets which activate JAK and STAT pathways leading to megakaryocyte growth and platelet production [21]. Production of TPO decreases in advanced liver disease, further contributing to thrombocytopenia [22]. Initial efforts to utilize recombinant TPO led to the production of cross-reactive antibodies in 10% of patients. Subsequent second-line TPO receptor agonists were developed to be structurally different from endogenous TPO and have thus evaded the complication of auto-antibodies [21].

Eltrombopag and romiplostim were the first second-generation TPO receptor agonists approved for the treatment of thrombocytopenia in hematologic disorders. However, application of these agents was limited in cirrhosis patients due to concern for portal vein thrombosis (PVT) [23]. However, avatrombopag and lusutrombopag were recently approved for the treatment of thrombocytopenia in patients with chronic liver disease undergoing elective procedures. In the global phase III ADAPT-1 and ADAPT-2 trials, patients with chronic liver disease were randomized to receive either 40mg or 60mg of avatrombopag based on their baseline platelet counts for a total of five days before undergoing an elective procedure [24]. A total of 435 patients were enrolled in both trials. The primary endpoint was the proportion of patients that did not require platelet transfusions for bleeding up to seven days after the procedure. In the ADAPT-1 trial, the primary endpoint was reached in 65.6% of patients who received 60mg avatrombopag and 88.1% of the patients who received 40mg dose compared to placebo (p <0.0001). Similarly, in ADAPT-2 trial, 68.6% patients reached primary endpoint in 60mg group and 87.9% of patients in 40mg group compared to 33.3-34.9% in placebo group (p < 0.001). In both trials, platelet counts generally peaked 10-12 days after the first administration and returned to baseline by day 35. The overall safety profile of avatrombopag was favorable as the risk of side effects was not significantly increased compared to the placebo group. However, it is unclear how to deduce the overall risk of development of PVT with avatrombopag as both trials did not include patients with a higher risk of developing PVT such as those with decreased portal vein velocity, prior history of thrombosis, or patients with advanced hepatocellular carcinoma [24].

Lusutrombopag is the most recent TPO receptor agonist approved in the United States for use in patients with advanced liver disease undergoing elective procedures. The FDA approval was based on two phase III trials, L-PLUS-1, and L-PLUS-2. The L-PLUS-1 trial was a double-blinded phase III trial of 96 patients in Japan in which the patients were randomized to receive once-daily lusutrombopag 3mg daily vs placebo for up to 7 days prior to the procedure. The primary end point was defined as the proportion of patients not requiring platelet transfusion before the invasive procedure with a target platelet count of >50K/uL [25]. The primary endpoint was reached in 79.2% of patients in lusutrombopag group compared to 12.5% in the placebo group (p<0.0001). Up to 50% of patients in the lusutrombopag group maintained peak platelet levels up to 10-17 days after the procedure. Similarly, in the L-PLUS-2 trial, 65% of patients in lusutrombopag group did not require platelet transfusion compared to 29% of patients in the placebo (p<0.0001) [26]. Rates of adverse events including PVT were not statistically significant compared to the placebo group. However, similar to avatrombopag trials, L-PLUS-1 and L-PLUS-2 trials also excluded patients with an advanced liver disease most at risk of developing portal vein thrombosis.

Given a relatively safe drug profile, TPO receptor agonists may be the preferred treatment of choice in improving thrombocytopenia for a short duration compared to standard platelet transfusions. While there are no absolute contraindications in chronic liver disease patients, it may be reasonable to avoid their use in certain patients such as those with prior history of thrombosis, pregnancy, or slow portal vein flow. The clinical efficacy of long-term use of TPO receptor agonists has not been well studied and as such, these drugs cannot be useful in improving thrombocytopenia for longer durations.

## Conclusions

Severe thrombocytopenia increases the risk of bleeding in patients with chronic liver disease and can often complicate or delay clinical care in these patients. However, no consensus currently exists for ideal platelet thresholds prior to surgical interventions and no specific guidelines exist for utilization of blood transfusion over other treatment strategies. While platelet transfusions are the most frequently utilized for the management of thrombocytopenia, the risks of transfusion reaction and possible alloimmunization limit their ability for prolonged/recurrent use. Therefore, other therapies ought to be utilized in higher frequency to avoid complications associated with platelet transfusions. Further research and subsequent establishment of guidelines is warranted to determine the best course of prophylactic treatment of thrombocytopenia in patients with chronic liver disease, including the use of TPO receptor agonists. 

## References

[1] Gauer RL, Braun MM (2012). Thrombocytopenia. Am Fam Physician.

[2] Afdhal N, McHutchison J, Brown R (2008). Thrombocytopenia associated with chronic liver disease. J Hepatol.

[3] Mitchell O, Feldman DM, Diakow M, Sigal SH (2016). The pathophysiology of thrombocytopenia in chronic liver disease. Hepat Med.

[4] Giannini E, Botta F, Borro P (2003). Relationship between thrombopoietin serum levels and liver function in patients with chronic liver disease related to hepatitis C virus infection. Am J Gastroenterol.

[5] Miller JB, Figueroa EJ, Haug RM, Shah NL (2019). Thrombocytopenia in chronic liver disease and the role of thrombopoietin agonists. Gastroenterol Hepatol (N Y.

[6] Harrison MF (2018). The misunderstood coagulopathy of liver disease: a review for the acute setting. West J Emerg Med.

[7] Nilles KM, Caldwell SH, Flamm SL (2019). Thrombocytopenia and procedural prophylaxis in the era of thrombopoietin receptor agonists. Hepatol Commun.

[8] Lange NW, Salerno DM, Berger K, Cushing MM, Brown RS Jr (2021). Management of hepatic coagulopathy in bleeding and nonbleeding patients: an evidence-based review. J Intensive Care Med.

[9] Massoud OI, Zein NN (2017). The effect of transjugular intrahepatic portosystemic shunt on platelet counts in patients with liver cirrhosis. Gastroenterol Hepatol (N Y.

[10] Alvarez OA, Lopera GA, Patel V, Encarnacion CE, Palmaz JC, Lee M (1996). Improvement of thrombocytopenia due to hypersplenism after transjugular intrahepatic portosystemic shunt placement in cirrhotic patients. Am J Gastroenterol.

[11] Karasu Z, Gurakar A, Kerwin B (2000). Effect of transjugular intrahepatic portosystemic shunt on thrombocytopenia associated with cirrhosis. Dig Dis Sci.

[12] Jabbour N, Zajko A, Orons P, Irish W, Fung JJ, Selby RR (1998). Does transjugular intrahepatic portosystemic shunt (TIPS) resolve thrombocytopenia associated with cirrhosis?. Dig Dis Sci.

[13] Ogata T, Okuda K, Sato T (2013). Long-term outcome of splenectomy in advanced cirrhotic patients with hepatocellular carcinoma and thrombocytopenia. Kurume Med J.

[14] Amin MA, el-Gendy MM, Dawoud IE, Shoma A, Negm AM, Amer TA (2009). Partial splenic embolization versus splenectomy for the management of hypersplenism in cirrhotic patients. World J Surg.

[15] Wu B-G, Chou AS-B, Hoo G-J LM-C (2017). Eighty percent partial splenic embolization is a safe and effective procedure in management of cirrhotic hypersplenism. Formos J Surg.

[16] Hayashi H, Beppu T, Okabe K, Masuda T, Okabe H, Baba H (2008). Risk factors for complications after partial splenic embolization for liver cirrhosis. Br J Surg.

[17] Hill A, Elakkad A, Kuban J (2020). Durability of partial splenic artery embolization on platelet counts for cancer patients with hypersplenism-related thrombocytopenia. Abdom Radiol (NY).

[18] Hadduck TA, McWilliams JP (2014). Partial splenic artery embolization in cirrhotic patients. World J Radiol.

[19] N'Kontchou G, Seror O, Bourcier V (2005). Partial splenic embolization in patients with cirrhosis: efficacy, tolerance and long-term outcome in 32 patients. Eur J Gastroenterol Hepatol.

[20] He XH, Gu JJ, Li WT (2012). Comparison of total splenic artery embolization and partial splenic embolization for hypersplenism. World J Gastroenterol.

[21] Bussel J, Kulasekararaj A, Cooper N, Verma A, Steidl U, Semple JW, Will B (2019). Mechanisms and therapeutic prospects of thrombopoietin receptor agonists. Semin Hematol.

[22] Peck-Radosavljevic M, Zacherl J, Meng YG (1997). Is inadequate thrombopoietin production a major cause of thrombocytopenia in cirrhosis of the liver?. J Hepatol.

[23] Dultz G, Kronenberger B, Azizi A (2011). Portal vein thrombosis as complication of romiplostim treatment in a cirrhotic patient with hepatitis C-associated immune thrombocytopenic purpura. J Hepatol.

[24] Terrault N, Chen YC, Izumi N (2018). Avatrombopag before procedures reduces need for platelet transfusion in patients with chronic liver disease and thrombocytopenia. Gastroenterology.

[25] Hidaka H, Kurosaki M, Tanaka H (2019). Lusutrombopag reduces need for platelet transfusion in patients with thrombocytopenia undergoing invasive procedures. Clin Gastroenterol Hepatol.

[26] Peck-Radosavljevic M, Simon K, Iacobellis A (2019). Lusutrombopag for the treatment of thrombocytopenia in patients With chronic liver disease undergoing invasive procedures (L-PLUS 2). Hepatology.

